# Patients’ perception of dignity in an Italian general hospital: a cross-sectional analysis

**DOI:** 10.1186/s12913-015-0704-8

**Published:** 2015-01-28

**Authors:** Paola Ferri, Jennifer Muzzalupo, Rosaria Di Lorenzo

**Affiliations:** School of Nursing, Department of Diagnostic, Clinical and Public Health Medicine, University of Modena and Reggio Emilia, via Del Pozzo n° 71, 41124 Modena, Italy; Servizio Psichiatrico Diagnosi e Cura, Department of Mental Health, Az-USL Modena, via Giardini n°1355, 41126 Modena, Italy

## Abstract

**Background:**

Dignity is related to a patient’s respect, privacy, information and autonomy. Maintaining dignity is defined as ethical goal of care. Although the importance of dignity has been widely recognized, there is limited research that investigates if dignity is really maintained in clinical practice and few studies have been conducted in acute hospital settings with adults across the age range. The aim of the study was to explore inpatients’ perception of dignity in an hospital setting.

**Methods:**

This descriptive cross-sectional study was carried out in 10 medical and surgical wards of a General Hospital in Modena (Italy). We collected a purposive sample of 100 patients by selecting 10 participants from each ward who met following criteria: hospitalized for more than three days, at least 18 years old, not mentally ill, willing to participate and able to speak Italian. We developed a 15-item anonymous questionnaire divided into three sections: “physical privacy”, “information and autonomy”, “nurse-patients respectful interaction”.

**Results:**

The percentages of positive (preserved dignity perception) were more frequent than negative (not preserved dignity perception) and no answers with a statistically significantly difference among the three sections (Pearson chi2 = 150.41, p < 0.0001). The frequency of positive or negative answers was statistically significantly related to the preservation of dignity according to the following questions (p < 0.005, multivariate logistic regression): “privacy to use the bathroom” and “respectful interaction”, as protective factors and “maintaining of body privacy”, “involvement in the care process”, “correct communication” as risk factors.

**Conclusions:**

Dignity was quite but not completely maintained according to the standards expected by patients. According to patients’ views, privacy of the body during medical procedures and respectful nurse-patient interactions were preserved more than information and verbal communication. Listening to patients’ views on the specific factors they consider useful to maintaining their dignity can help in this process. Recognizing and focusing on these factors will help professionals to establish practical measures for preserving and promoting patients' dignity and providing more dignified care. Dignity should be extensively and systematically pursued as other important clinical goals.

**Electronic supplementary material:**

The online version of this article (doi:10.1186/s12913-015-0704-8) contains supplementary material, which is available to authorized users.

## Background

Dignity is one of the fundamental human rights [1]. The word is derived from two Latin words: ‘*dignitas*’, which means merit, implying that one has to achieve something to be dignified; and ‘*dignus*’ [2,3], which means worth, suggesting a quality that renders something valuable or confers value for one’s wealth [4]. Most authors have claimed that disease can reduce a person’s ability to maintain privacy and dignity, although all people desire to maintain dignity properly even in adverse situations [5-7]. This need is especially pertinent to acute hospital settings [8,9], as endorsed by the Amsterdam declaration on the promotion of patients’ rights [10], which declared that “Patients have the right to be treated with dignity, which should be rendered with respect for their culture and values” [11]. Dignity in the caring process contains the values of “autonomy, truth, justice, and responsibility to human rights” [12] and is demonstrated by “attentiveness, awareness, personal respect, engagement, fraternity and an active defense of the patient” [13,14]. Baillie asserted that preserving human dignity is integral to the caring mission of nursing [9] and she recognized that health problems may threaten dignity (thereby leading to ‘indignity’). Respecting human rights and maintaining dignity were also defined as ethical goals of nursing care [15], which should not differ because of a patient’s race, age, religion, sickness or handicap, gender, or political, social and economic status [16,17].

The need to preserve the dignity of patients is included in most policy statements for health professionals [5,13] and the significance of patient dignity is also reflected in various professional nursing bodies’ codes of conduct [4,17]. The third article of the Italian Nursing Code of Ethics (2009) is referred to human dignity: “*The responsibility of nurses consists of treating and caring people in respect of life, health, freedom and dignity of each person*” [18].

The American Nurses Association states: “*The nurse, in all professional relationships, practices with compassion and respect for the inherent dignity, worth and uniqueness of every individual, unrestricted by considerations of social or economic status, personal attributes, or the nature of health problems*” [19]. The same statements are present in other nursing code as in the Canadian Nurses Association (“*Nurses support the person … receiving care in maintaining their dignity and integrity*..”) [20] and the Nursing and Midwifery Board of Australia (“*Nurses actively preserve the dignity of people through practiced kindness and by recognizing the vulnerability and powerlessness of people in their care…*”) [21].

In 2008, the Royal College of Nursing (RCN), a professional body representing UK Nurses, launched a campaign, “Dignity at the heart of everything we do”, and proposed a definition of dignity to underpin the campaign: “Dignity is concerned with how people feel, think and behave in relation to the worth or value of themselves and others. To treat someone with dignity is to treat them as being of worth, in a way that is respectful of them as valued individuals” [22].

The International Council of Nurses proposed a code of ethics for nurses, which includes four elements: 1) “Nurses and people”, which represents the nurses’ respect for their patients’ race, age, religion, sickness or handicap, gender, political, social and economic status; 2) “Nurses and practice”, which suggests that nurses may offer competent care by enhancing their professional care abilities; 3) “Nurses and the profession”, which indicates that nurses should develop professional knowledge based on research; 4) “Nurses and co-workers”, which refers to nurses acting as mediators amongst various health care professional groups and protecting patients and their family members if endangered by co-workers [16].

Dignity matters in healthcare are important for patients, families and professionals [13]. It is now widely recognized that quality of care is dependent not only on the treatment received, but also on the way the care is delivered [23]. In fact, the lack of dignity may lead to poorer health outcomes for patients [8] and could influence patients’ recovery [2].

Nurses are responsible for fostering human dignity through their interactions with patients and with others on the health-care team [17]. It has also been suggested that if nurses do not feel that their dignity is respected by colleagues and the organization they work for, this may impact on the patient experience [16]. A WHO investigation in 41 countries highlighted that most participants selected dignity as the second most important domain, behind prompt access to care [24]. A recent meta-synthesis has highlighted that recognition of nurse professional dignity could have a positive impact on respect for patients’ dignity, since good care and adequate relationships with patients can be expected from respected and professionally valorized nurses [25].

Although respecting human dignity is an essential element in health care settings, there are different factors affecting patients’ dignity that should be considered, such as effective communication, maintenance of privacy and physical environment, protecting patients, providing privacy, preserving autonomy and sense of control, forms of address, providing adequate information [2,4,8,9,11,23,26-29], staff’s decency [4], satisfying patients’ needs [2], staff’s sense of humor [8,9] and taking into account patients’ opinions [4,8,9,16,26,28]. Most authors highlighted that patients have identified the importance of dignity in care and their perceptions of respectful and dignified treatment is closely related to their satisfaction with health care [6,9,22].

### Privacy

Several papers have explored privacy and the link between privacy and dignity [8,23,27,29-31]. Although, privacy is a basilar health care right, there is no universal definition of privacy [30,32]. Scott et al. [30] suggested that four components are inherent in the concept of privacy: physical, psychological, social and informational. Some or all of these aspects of privacy may be important to maintaining dignity in hospital care. Most studies investigated the area of physical privacy, focusing on the hospital environment and on the most common problems, such as noise, limited space and restrictions [11,27,32]. Some authors defined personal space as: “an invisible boundary surrounding the self; intrusion into this space creates tension or discomfort” [30]. Informational privacy can be defined as the individual’s right to determine how, when and to what extent information regarding himself may be given to other persons or organizations [30,33]. Protection of patient privacy, in all its dimensions, can be difficult in a busy hospital environment [30] and could represent a paradox in shared hospital room situations. Informational privacy often has to do with the confidentiality of patient information, which means that this aspect is bound to take on increasing importance in the future with the continuing growth of computerized medical records [32]. Nursing interventions to maintain personal space will help the patient demonstrate control of his or her space and facilitate adaptation to the environment. Health professionals will also provide physical barriers to protect the patient’s personal space when there is a need for close contacts and promote the patient’s trust in professionals. All these interventions can also help to promote privacy [32]. The respect for privacy and personal dignity are interconnected, as Woogara stated: “privacy is intrinsic to the individual’s physical, mental, emotional and spiritual well-being” [27].

### Autonomy

The term of “autonomy” is used with the following meanings in health care: self-determination, self-rule, liberty of rights, freedom of will; and being one’s own person [30,32]. Autonomy depends on the patient’s ability to make independent choices, on his adequate knowledge and on correct information received. Professionals must first help the patient to become comfortable in his surroundings, inform him concerning available options, and empower the patient with sufficient knowledge to exercise confident (self-efficacious) control. Information is an important element of autonomous control; but unless the patient has confidence and competence to understand, it provokes stress rather than providing comfort [30,34].

### Information and consent

A further element of respect for autonomy in the healthcare context is seeking the individual’s consent before interventions and treatments. Providing and seeking information and facilitating involvement in informed decision-making are considered elements of the informed consent process. One cannot exercise one’s autonomy without the relevant information and support for input into decision making. Information is central to informed consent [30]. To enable patients to give informed consent they must receive adequate information, understand this information and consent voluntarily. Most guidelines have detailed the process of administering the informed consent and, in particular, have underlined the importance of both the patients’ understanding of the information and the patients’ freedom and competence to make a decision. The patients’ signature on the informed consent form represents the conclusion of a complete discussion between physician/professional and patient. Autonomy and informed consent are interlinked, as you cannot comply with one without complying with the other. In fact, giving consent before any healthcare procedure is a vital expression of the patient’s autonomy. It is, of course, also recognition of the patient’s right to privacy. Invasion of a personal space or body for treatment or investigation without the patient’s consent is a legal and moral wrong [30]. There are few empirical studies investigating the degree to which patients are informed participants in the consent process [32]. Two small studies reported in the UK literature suggested that there is significant work to be done before we can be confident that patients really understand what they are consenting to and what the potential outcomes of the proposed treatment are [30]. Although the importance of dignity has been widely recognized, there is limited research that investigates if dignity is really maintained in clinical practice [4] and few studies have been conducted in acute hospital settings with adults across the age range [9,17]. An Italian qualitative survey, which explored the meaning of dignity and the related variables in a hospital setting, highlighted 3 main components that favored or reduced dignity: “factors related to the patient, to the physical environment or to the hospital staff” [35]. In this regard, other Italian authors suggested that the assessment of patients’ dignity perception in many different hospital settings is necessary to implement ethically correct health procedures [36].

The aim of this study was to explore inpatients’ perception of dignity in an Italian General Hospital setting through the administration of an anonymous questionnaire based on the main factors of dignity, in accordance with the Italian Nursing Code of Ethics: physical privacy, respect, information and autonomy.

## Methods

### Design

This descriptive cross-sectional study was conducted from 1st to 31th July 2012.

### Setting and sample

The research was carried out in the following medical and surgical wards of a General Hospital in Modena (Italy): Cardiology, Gastroenterology, Pulmonology, Infectious Diseases, Internal Medicine, Nephrology, Orthopedics-Traumatology, Hand Surgery, Transplant Surgery, Thoracic Surgery. To be included in the study, patients had to meet the following criteria: hospitalized for more than three days, at least 18 years old, not mentally ill, willing to participate in the study and able to speak Italian. From each medical and surgical ward, 10 participants who met these criteria were selected in order to collect a purposive sample of 100 patients. The demographic data of our sample are shown in Table 1.

**Table 1:** **Demographic data (no. = 100 patients)**

**Variables**		**Total (n %)**
**Gender**	M	48 (48%)
	F	52 (52%)
**Age**	18-29	8 (8%)
	30-39	17 (17%)
	40-49	19 (19%)
	50-59	24 (24%)
	60-69	18 (18%)
	70-79	8 (8%)
	80-89	6 (6%)
**Nationality**	Italian	96 (96%)
	Extra-EU	4 (4%)
**Duration of hospitalization (weeks)**	≤1	38 (38%)
	>1 to ≤2	20 (20%)
	>2 to ≤3	16 (16%)
	>3 to ≤4	12 (12%)
	>4	14 (14%)

### Materials

The questionnaire was developed, in accordance with the Italian Nursing Code of Ethics (2009) [18] and literature [4,8,9,37], in order to highlight the patients’ perception of dignity by evaluating three main topics: “physical privacy”, “information and autonomy”, “respectful nurse-patient interaction”. Before being administered to patients, the questionnaire was submitted to 8 experts and was modified according to their comments. The comprehensibility of the questionnaire was tested by administering them to 10 patients. Finally 15 questions were chosen and divided into three sections relative to the main topics: I) questions 1 to 5 concerning “physical privacy”, II) questions 6 to 9 concerning “information and autonomy”, III) questions 10 to 15 concerning “nurse-patients respectful interaction”. The 15 questions, as shown in Table 2, required dichotomous answers (YES/NO). The questionnaire was anonymously administered. Additional files show this in more detail [see Additional files 1 and 2].

**Table 2:** **Questions and answers concerning “dignity”**

**No.**	**Questions**	**Positive answers (%)**	**Negative answers (%)**	**No answers (%)**
1	“Before you exposed the private parts of your body in order to undergo medical procedures, had nurses closed the door of your room?”	66%	10%	24%
2	“Did you receive enough privacy when you needed to use the bed-pan and/or urine bottle to urinate, e.g. did nurses cover you with a bed sheet or blanket?”	46%	14%	40%
3	“Did nurses take care to cover the private parts of your body at the end of each procedure?”	74%	4%	22%
4	“Did you have privacy to use the bathroom?”	85%	5%	10%
5	“While undergoing medical procedures which required the exposure of private parts of your body, did the door of your room remain closed?”	72%	8%	20%
6	“Did nurses ask your permission before performing care procedures on your body?”	67%	19%	14%
7	“Did nurses provide information on the diagnostic and therapeutic procedures that you needed?”	62%	30%	8%
8	“Did the nurses involve you in your health program and allow you to make decisions in this regard?”	46%	44%	10%
9	“Did nurses let you do daily activities (bathing, dressing, feeding) if you were able to perform them by yourself?”	61%	20%	19%
10	“Did nurses introduce themselves to you at your first meeting in hospital?”	24%	74%	2%
11	“Did nurses ever refer to you using respectful language without calling you by nicknames?”	88%	8%	4%
12	“Did nurses treat you with respect without using excessively familiar manner?”	77%	20%	3%
13	“When talking to other health care professionals, did nurses refer to you using your name rather than the number of your bed ?”	76%	10%	14%
14	“Did nurses interact with you using a kind and warm tone?”	75%	25%	0%
15	“During the discussion of personal matters, did nurses ensure sufficient privacy?”	74%	22%	4%

### Statistical analysis

The percentage of positive (dignity preserved) and negative (dignity not preserved) answers was analyzed (chi2 test) and correlated to the questions, in order to identify the risk or protective factors related to dignity (multivariate logistic regression). Data were statistically analyzed through STATA program.

### Ethical considerations

This study was performed in accordance with the Declaration of Helsinki and was authorized by both the Medical Director and Nurse Manager of “Policlinico”, the University Hospital of Modena. It was approved by the ethics board of local Nurses Association. Before administering the questionnaire, the researcher explained to each participant the purpose and significance of this study, as well as the participant’s right to withdraw from it at any time without any impact on clinical treatment. Each patient received verbal and written information from the main researcher. The anonymity and confidentiality of participants were assured and their decision to participate voluntarily in this study was respected. All patients signed an “informed consent” form before being included in the study.

## Results

Most patients in our sample were Italians, with a normal age distribution and an equal number of males and females. The majority of them (38%) was hospitalized for ≤1 week (Table 1).

**Figure 1:**
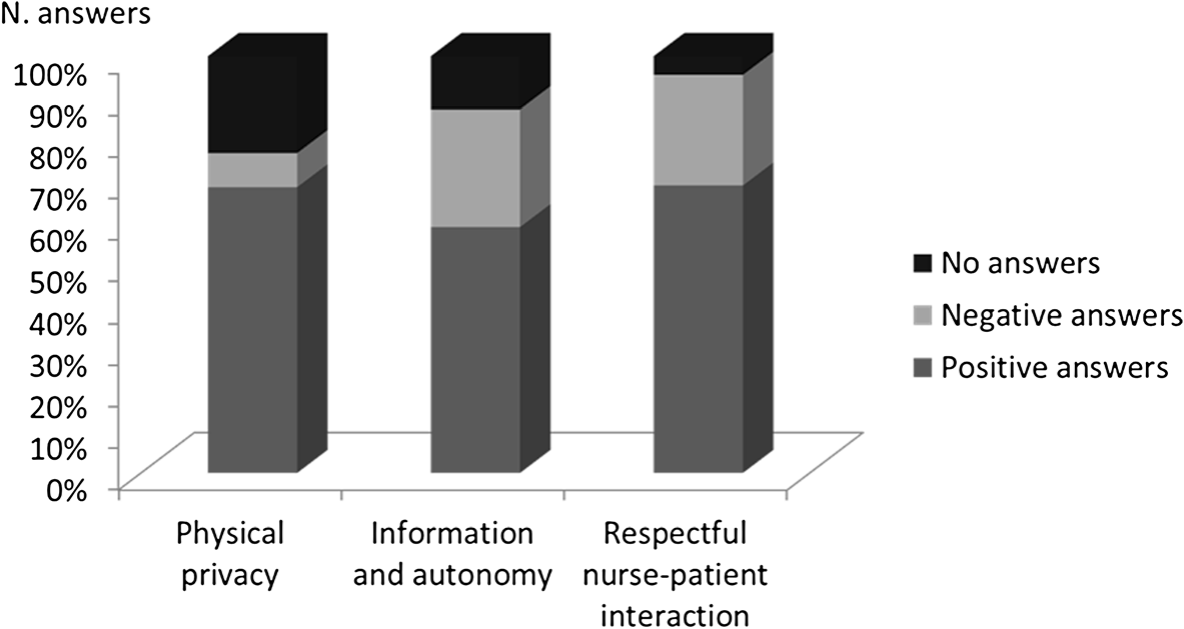
**Answers to the questions concerning dignity.**

In Table 2, the percentages of positive, negative and no answers to the 15 questions are shown. Positive answers (preserved dignity perception) were more frequent than negative answers (not preserved dignity perception) and no answers, with a statistically significant difference among the three sections (Pearson chi2 = 150.41, p < 0.0001, chi2 test) (Figure 1).

In particular:

- for “physical privacy”, positive answers (68.6%) and no answers (23.2%) were more frequent than negative answers (8.2%);
- for “information and autonomy”, positive (59%) were more numerous than negative answers (28.25%) and no answers (12.75%);
- for “respectful nurse-patient interaction”, positive (69%) and negative (26.5%) answers were prevalent in comparison to no answers (4.5%).

The multivariate logistic regression analysis highlighted that the frequency of positive (preserved dignity perception) or negative (not preserved dignity perception) answers to 5 questions was statistically significantly related to the preservation of dignity (p < 0.005, p < 0.0001), as:

- protective factors, question no.4 regarding “privacy in bathroom” (“Did you have privacy to use the bathroom?”) and question no.11 regarding “respectful interaction” (“Did nurses ever refer to you using respectful language without calling you by nicknames?”);
- risk factors, questions no.2,8 and 10, concerning, respectively, maintaining body privacy (“Did you receive enough privacy when you needed to use the bed-pan and/or urine bottle to urinate, e.g., did nurses cover you with a bed sheet or blanket?”), involvement in the care process (“Did the nurses involve you in your health program and allow you to make decisions in this regard?”) and correct communication between patient-professional (“Did nurses introduce themselves to you at your first meeting in hospital?”) (Table 3).

**Table 3:** **Multiple logistic regression analysis of dignity perception**

**No.**	**Questions**	**Odds ratio**	**Standard error**	**p**	**95% Confidence interval**
2	“Did you receive enough privacy when you needed to use the bed-pan and/or urine bottle to urinate, e.g., did nurses cover you with a bed sheet or blanket?”	2.288	.663	0.005	1.288 - 4.033
4	“Did you have privacy to use the bathroom?”	.342	.120	0.005	.172 - .681
8	“Did the nurses involve you in your health program and allow you to make decisions in this regard?”	2.288	.663	0.005	1.287 - 4.033
10	“Did nurses introduce themselves to you at your first meeting in hospital?”	6.147	1.938	0.0001	3.314 - 1.403
11	“Did nurses ever refer to you using respectful language without calling you by nicknames?”	.081	.045	0.0001	.027 - .239

## Discussion

This study, which evaluated patients’ perception of respect for their dignity during hospitalization in medical and surgical wards in a general hospital, highlighted that “dignity” was quite but not completely maintained according to the standards expected by patients and confirmed the results of earlier research [4].

All the participants were keen to discuss issues regarding promotion of dignity and their participation was good, as evidenced by the low percentage of no answers. The difference in no answers can be due to the kind of question, not fitting the clinical situation of patients (as some questions concerning physical privacy) or to difficulty in understanding the issue. The section of questions concerning “nurse-patients respectful interaction” obtained the lowest frequency of no answers, perhaps due to the universality of this topic, easily understood by everyone. The percentage of positive answers evidenced that, according to patients’ views, privacy of the body during medical procedures and respectful nurse-patient interactions were preserved more than information and verbal communication. In particular, privacy to use the bathroom was frequently maintained, whereas body privacy in some medical procedures which required the body to be exposed was not completely preserved. Avoidable body exposure can be considered an important aspect of dignity, as most studies reported [4,8,9,23].

Most interactions between patient-professional were characterized by respect, kind and warm attitude, according to the highest number of positive answers obtained in our questionnaire on this topic, whereas the information and autonomy questions received the lowest number of positive answers. This data suggests that valid health communication in order to permit the patient to give an informed consent or to make health care decisions is up to now lacking or insufficient, as most studies have highlighted [30]. The framework that our study outlines is characterized by a good quality of interaction based on good acceptance of the patient: this attitude provides attention and satisfaction of basic needs by means of non-verbal interaction like a “maternal code” attitude permits. On the contrary, the clear verbal information necessary to improve the patients’ autonomy, like a “paternal code” which fosters the application of rules, is the most deficient aspect as revealed by this research. We can indirectly infer that hospitalization can induce such a regression of autonomy as to require care for elementary needs, which can be satisfied by a behavior of complete acceptance and support (the so called *maternage* or *maternal holding* according to psychoanalytic authors) [38]. Otherwise, in order to avoid dependence on the institution which each hospitalization, especially for long term, can induce, a good relationship with patients can be essential for rapid recovery and discharge as well as for good outpatient care. This relationship should be based on verbal and clear communication and information in order to promote patients’ awareness and responsibility for their treatments, thus leading the patient to making decisions about his/her care programs.

We suggest that the results of our study focused on nurse-patient interactions can be extended to all health professionals in all hospital settings, since dignity in care is a universal need of each patient in all phases of diagnostic, therapeutic and rehabilitative programs. We infer that more careful attention to dignity permits patients (and their family or caregiver) to better tolerate the discomfort of physical diseases and the secondary psychological vulnerability, especially in cases of severe illness with burdensome disability. Therefore, the procedures aimed at preserving dignity can be considered not only ethical measures but also efficacious modalities to ameliorate both the therapeutic compliance of patients and the relationship between patient and health professionals [39].

In this regard, one of the greatest scholars on this topic, Chochinov [40], coined the expression of “dignity therapy” to indicate the ethical end-of-life-treatments which help terminally ill patients to cope with their emotional feelings of death.

The areas investigated by our study, especially respect for the patient and clarity in communication, overlap the 4 basic dimensions of dignity named A (attitude), B (behavior), C (compassion) and D (dialogue), indicated by Chochinov [41,42] as the most relevant areas for maintaining dignity of patients and, at the same time, for teaching best clinical practice to all health professions.

The preservation of dignity in the areas of physical privacy, communication of information, autonomy and respectful interaction with patient should be the basic foundations of each health treatment and care activity. All professionals of the health staff (physicians, nurses, nursing assistants, etc.) have to maintain dignity in care and treatment not only to guarantee an ethical approach but also to obtain the clinical improvement of patients. Nevertheless, nurses are closer than other professionals to the patients because the care they provide addresses the whole person, for many hours a day, in intimate and delicate psychological and physical zones.

### Limitations and future development

This research has some methodological limits: the sample was restricted, not randomized and was only representative of the patients admitted in surgical and medical wards of a single hospital in northern Italy. In this regard, we have to point out that, in Modena, patients’ perception about the maintenance of their dignity during hospitalization has not been completely analyzed. This study represents innovative research on this topic. Another important limitation is represented by the questionnaire, which should be further validated by other researchers in other scenarios. This preliminary study is only a validation attempt of a survey instrument to evaluate dignity in an acute hospital setting which, up to now, has not been available since the only questionnaire in Italian is Patient Dignity Inventory [43], validated only in terminally ill out-patient settings [44].

Further steps should be taken to ensure that various guidelines on dignity are fully implemented [4] and other instruments for dignity evaluation should be implemented in order to study this topic at the national level in different health settings. Listening to patients’ views on the specific factors they consider useful to maintaining their dignity can help in this process. Recognizing and focusing on these factors will help professionals to establish practical measures for preserving and promoting patients' dignity and providing more dignified care [2,16]. Although long established traditions and cultures embedded within institutions could render it difficult for staff to promote dignity, we suggest, in accordance with other authors [5,8,37], that dignity should be considered as important as other clinical goals to be extensively and systematically pursued.

## Conclusions

We conclude, in accordance with other authors [2,9], that, whereas the hospital environment should provide the physical and managerial facilities for promoting patients’ dignity, each individual staff member must promote patients’ dignity through their own behavior and must be aware of their impact on patients’ vulnerability.

We underline that “dignity conserving care”, which has an important effect “on how patients experience illness” [40], is mostly influenced by the capacity of professionals to empathize with the condition of patients and can represent the quality of patient-health professional interaction. The safeguarding of a patient’s dignity can promote not only a greater “emotional comfort” [45] or “a sense of well-being”, but it can be a necessary and unavoidable premise for recovery due to reduced risk for institutional regression and dependence. Therefore, we suggest that the respect for patients’ dignity is not only an ethical goal in patient care but it can represent one of the positive factors which favors the process of recovery.

## Additional files

Additional file 1:
**Questionario italiano sul rispetto della dignità (Italian version).**
Additional file 2:
**Patients’ perception of dignity Italian Questionnaire (English version).**
