# Supplementary material for: Patients’ perception of dignity in an Italian general hospital: a cross-sectional analysis
Source: BMC Health Serv Res. 2015 Jan 28;15:41. doi: 10.1186/s12913-015-0704-8 (PMC4312597; doi:10.1186/s12913-015-0704-8)
Supplement: Additional file 1: — Questionario italiano sul rispetto della dignità (Italian version). [file 12913_2015_704_MOESM1_ESM.doc]

**Questionario italiano sul rispetto della dignità**

Gentile Sig./Sig.ra le chiediamo la cortesia di rispondere alle domande elencate, con riferimento all’attuale ricovero ospedaliero, scegliendo tra le opzioni SI e NO

| 1 | *“Prima di effettuare procedure che comportavano l’esposizione di parti intime del suo corpo, gli infermieri hanno chiuso la porta della stanza di degenza?”* | **SI** | **NO** |
| --- | --- | --- | --- |
| 2 | *“Le è stata data sufficiente privacy durante l’utilizzo della padella/pappagallo (ad esempio, coperta sulle parti intime del suo corpo)?”* | **SI** | **NO** |
| 3 | *“Gli infermieri avevano l’accortezza di ricoprire, al termine di ogni procedura, le parti intime del suo corpo che avevano scoperto per svolgere la procedura stessa?”* | **SI** | **NO** |
| 4 | *“Aveva la possibilità di usare il bagno mantenendo la sua privacy?”* | **SI** | **NO** |
| 5 | “*Durante l’esecuzione, da parte degli infermieri, di procedure che richiedevano l’esposizione di parti intime del suo corpo, la porta della sua camera di degenza rimaneva chiusa?”* | **SI** | **NO** |
| 6 | *“Gli infermieri le hanno chiesto il consenso prima di svolgere procedure assistenziali sul suo corpo?”* | **SI** | **NO** |
| 7 | *“Gli infermieri le hanno fornito informazioni sulle procedure diagnostiche e terapeutiche che hanno effettuato su di lei?”* | **SI** | **NO** |
| 8 | *“Gli infermieri la coinvolgevano nel suo programma di assistenza, permettendole di prendere decisioni?”* | **SI** | **NO** |
| 9 | *“Gli infermieri le lasciavano svolgere quelle attività che era in grado di fare autonomamente (lavarsi, vestirsi, alimentarsi)?”* | **SI** | **NO** |
| 10 | *“In occasione del vostro primo incontro, gli infermieri si sono presentati con il loro nome?”* | **SI** | **NO** |
| 11 | *“Gli infermieri hanno interpellato la sua persona in modo corretto senza utilizzare dei nomignoli?”* | **SI** | **NO** |
| 12 | *“Gli infermieri si sono rivolti a lei in modo rispettoso, senza darle del tu?”* | **SI** | **NO** |
| 13 | *“Gli infermieri hanno parlato di lei con altri operatori sanitari utilizzando il numero del suo letto per identificarla, invece del suo nome?”* | **SI** | **NO** |
| 14 | *“Gli infermieri si sono rivolti a lei con un tono gentile e accogliente?”* | **SI** | **NO** |
| 15 | *“Durante la discussione di argomenti personali, gli infermieri le hanno garantito una sufficiente riservatezza?”* | **SI** | **NO** |
