# Supplementary material for: Patients’ perception of dignity in an Italian general hospital: a cross-sectional analysis
Source: BMC Health Serv Res. 2015 Jan 28;15:41. doi: 10.1186/s12913-015-0704-8 (PMC4312597; doi:10.1186/s12913-015-0704-8)
Supplement: Additional file 2: — Patients’ perception of dignity Italian Questionnaire (English version). [file 12913_2015_704_MOESM2_ESM.doc]

**Patients’ perception of dignity Italian Questionnaire (English version)**

Dear Mr/Ms, we would like to know your perception of dignity in the current hospitalization. Therefore, we ask you to answer the following questions “YES or NO”.

| 1 | *“Before you exposed the private parts of your body in order to undergo medical procedures, had nurses closed the door of your room?”* | **YES** | **NO** |
| --- | --- | --- | --- |
| 2 | *“Did you receive enough privacy when you needed to use the bed-pan and/or urine bottle to urinate, e.g. did nurses cover you with a bed sheet or blanket?”* | **YES** | **NO** |
| 3 | *“Did nurses take care to cover the private parts of your body at the end of each procedure?”* | **YES** | **NO** |
| 4 | *“Did you have privacy to use the bathroom?”* | **YES** | **NO** |
| 5 | *“While undergoing medical procedures which required the exposure of private parts of your body, did the door of your room remain closed?”* | **YES** | **NO** |
| 6 | *“Did nurses ask your permission before performing care procedures on your body?”* | **YES** | **NO** |
| 7 | *“Did nurses provide information on the diagnostic and therapeutic procedures that you needed?”* | **YES** | **NO** |
| 8 | *“Did the nurses involve you in your health program and allow you to make decisions in this regard?”* | **YES** | **NO** |
| 9 | *“Did nurses let you do daily activities (bathing, dressing, feeding) if you were able to perform them by yourself?”* | **YES** | **NO** |
| 10 | *“Did nurses introduce themselves to you at your first meeting in hospital?”* | **YES** | **NO** |
| 11 | *“Did nurses ever refer to you using respectful language without calling you by nicknames?”* | **YES** | **NO** |
| 12 | *“Did nurses treat you with respect without using excessively familiar manner?”* | **YES** | **NO** |
| 13 | *“When talking to other health care professionals, did nurses refer to you using your name rather than the number of your bed ?”* | **YES** | **NO** |
| 14 | *“Did nurses interact with you using a kind and warm tone?”* | **YES** | **NO** |
| 15 | *“During the discussion of personal matters, did nurses ensure sufficient privacy?”* | **YES** | **NO** |
